# *BRCA2* Haploinsufficiency in Telomere Maintenance

**DOI:** 10.3390/genes13010083

**Published:** 2021-12-28

**Authors:** Soffía R. Gunnarsdottir, Hördur Bjarnason, Birna Thorvaldsdottir, Felice Paland, Margrét Steinarsdottir, Jórunn E. Eyfjörd, Sigrídur K. Bödvarsdottir

**Affiliations:** 1 BioMedical Center, School of Health Sciences, Faculty of Medicine, University of Iceland, 101 Reykjavík, Iceland; soffiarung@gmail.com (S.R.G.); hordurb123@gmail.com (H.B.); birnathorv@gmail.com (B.T.); felice.paland@student.uni-tuebingen.de (F.P.); jorunne@hi.is (J.E.E.); 2 Faculty of Medicine, Eberhard Karls University, 72074 Tübingen, Germany; 3 Department of Genetics and Molecular Medicine, Landspitali the National University Hospital of Iceland, 101 Reykjavík, Iceland; margret.steinarsdottir@gmail.com

**Keywords:** *BRCA2*, telomere, haploinsufficiency, Fanconi anemia, chromosomal instability

## Abstract

Our previous studies showed an association between monoallelic *BRCA2* germline mutations and dysfunctional telomeres in epithelial mammary cell lines and increased risk of breast cancer diagnosis for women with *BRCA2 999del5* germline mutation and short telomeres in blood cells. In the current study, we analyzed telomere dysfunction in lymphoid cell lines from five *BRCA2 999del5* mutation carriers and three Fanconi Anemia D1 patients by fluorescence in situ hybridization (FISH). Metaphase chromosomes were harvested from ten lymphoid cell lines of different *BRCA2* genotype origin and analyzed for telomere loss (TL), multitelomeric signals (MTS), interstitial telomere signals (ITS) and extra chromosomal telomere signals (ECTS). TL, ITS and ECTS were separately found to be significantly increased gradually between the *BRCA2*^+/+^, *BRCA2*^+/-^ and *BRCA2*^-/-^ lymphoid cell lines. MTS were found to be significantly increased between the *BRCA2*^+/+^ and the *BRCA2*^+/-^ heterozygous (*p* < 0.0001) and the *BRCA2*^-/-^ lymphoid cell lines (*p* < 0.0001) but not between the *BRCA2* mutated genotypes. Dysfunctional telomeres were found to be significantly increased in a stepwise manner between the *BRCA2* genotypes indicating an effect of BRCA2 haploinsufficiency on telomere maintenance.

## 1. Introduction

The breast cancer susceptibility 2 (*BRCA2*) gene was identified in 1995 and has been shown to play an essential role in DNA double strand break (DSB) repair by homologous recombination (HR) and DNA crosslink repair by the Fanconi anemia (FA) pathway [1,2]. Germline mutations in the *BRCA2* gene are known to be highly associated with the risk of diagnosis with breast, ovarian, prostate, and pancreatic cancer [3,4,5,6]. Breast cancer risk is not only associated with women but also men. In Iceland, a *BRCA2 999del5* truncation mutation in exon 9 was isolated in a family with several cases of male and female breast cancer [3]. This mutation has been shown to have a high frequency (0.7%) in the Icelandic population [5,7]. Cloning of the *999del5* mutant and expression studies showed that even though the short mutant RNA is produced there is no detectable corresponding protein, indicating *BRCA2* heterozygosity [8].

Multiple chromosomal abnormalities have been found in breast tumors with the *BRCA2 999del5* mutation [9,10] and in murine cells deficient in the *BRCA2* homolog [11]. The abnormalities include broken chromosomes and chromatids, markers of defective mitotic recombination, and tri- and quadriradial chromosomes that are known to be related to defects in the FA pathway [12]. Indeed, *BRCA2* has been shown to be one of the genes playing a key role in the FA pathway, subtype D1 [13]. Patients with the FA-D1 subtype have biallelic *BRCA2* germline mutations, whereas at least one of the mutations is mild, and has high susceptibility for diagnosis with leukemia, solid tumor, brain tumor and Wilm’s tumor in childhood [14].

Studies on the role of the BRCA2 protein have shown the requirement of HR-dependent RAD51 filament formation plus stabilization by BRCA2 and the core FA pathway for efficient protection in replication forks from degradation following short-term replication arrest. FA and BRCA2 defective cells are defective in fork protection and show an increase in genome instability, manifesting chromatid breaks and radial structures on mitotic chromosome spreads [15,16]. DNA replication of repetitive sequences like telomeres is known to take place in the late S-phase of the cell cycle. It has been shown that BRCA2 is required to limit replication stress (RS) at telomeres by RAD51 filament loading [17,18]. Our study on *BRCA2* heterozygous mammary epithelial cell lines showed telomere dysfunction such as chromosome end-to-end fusions, interstitial telomere sequences (ITS), telomere loss (TL), extrachromosomal telomere sequences (ECTS) and frequent telomere sister chromatid exchanges (T-SCE) as seen in cells that rely on the alternative lengthening of telomeres (ALT) in absence of telomerase. These results indicated the important role of BRCA2 in telomere stabilization and protection [9]. A recent study on our cohort showed that women with the *BRCA2 999del5* germline mutation and relative short telomere length measured in blood cells were at significantly higher risk of being diagnosed with breast cancer [19]. These results indicate that monoallelic *BRCA2* gene expression may not be enough to fulfill telomere maintenance, indicating *BRCA2* haploinsufficiency. Anticipation effects have also been reported from this study cohort where daughters of mother-daughter pairs with the *BRCA2 999del5* germline mutation have been shown to be diagnosed with breast cancer on average 10 years younger than the mothers [7].

The 50 years old Knudson’s two-hit hypothesis suggests that both alleles of a tumor suppressor gene require to be inactivated to cause tumor formation, as was first described in heritable cases with retinoblastoma [20]. We have, however, shown that only about half of the *BRCA2 999del5* breast cancer cases [21,22] and pancreatic cancer cases [23] had lost their *BRCA2* wild-type allele in the tumor tissue. Similar results have been reported from another study cohort where the absence of *BRCA2* locus-specific loss of heterozygosity was observed in 46% of breast tumors [24]. Of note is that *BRCA2 999del5* breast cancer cases with wild-type allelic loss had significantly worse breast cancer specific survival compared to cases that remained the wild-type allele [22]. A recent study showed the *BRCA2* haploinsufficient phenotype in vivo in breast tissues of *BRCA2* mutation carriers exhibiting DNA damage that resulted from failed RS- and DNA damage responses and consequently aneuploidy [25].

Mechanisms shown to respond to RS that could counteract karyotypic diversity and contribute to tumor progression include polymerase theta-mediated end joining (TMEJ) alternative repair pathway at resected DSBs when HR is deficient to repair broken forks [26,27]. Other recent studies have shown that genic RS induced by the absence of Brca2 led to delays in replication and mitotic DNA synthesis (MiDAS) [25,28]. RAD52 has been shown to promote MiDAS following RS that occurs independently of RAD51 [29]. Abrogation of Brca2 has been shown to reinforce MiDAS related break-induced replication (BIR) and engagement with the ALT pathway. For this Brca2-deficient ALT induction, Rad51 filament loading was dispensable, but Mre11 dependent DSB resection and Rad52 were required [30]. RS at telomeres has as well been linked to MiDAS [31,32].

In the present study, we compared telomere dysfunction in lymphoid cell lines with different *BRCA2* genotypes including two *BRCA2* wild-type cell lines, five *BRCA2* heterozygous cell lines with the *999del5* germline mutation and three FA-D1 cell lines with biallelic *BRCA2* mutations. The originality of this study was to compare all three *BRCA2* genotypes in a cell type not associated with *BRCA2* hereditary cancer. Results showed increased telomere abnormalities between the *BRCA2* genotypes in a stepwise manner, including TL, ITS and ECTS.

## 2. Materials and Methods

### 2.1. Lymphoid Cell Lines

Viable lymphoid cells from women with and without the heritable *BRCA2 999del5* mutation were Epstein-Barr virus transformed (Table 1). This was carried out according to permits from the Icelandic Data Protection Commission (2006050307) and Bioethics Committee (VSNb2006050001/03-16). Three FA-D1 lymphoid cell lines were generously provided by Professor Helmut Hanenberg, University of Duisburg-Essen, Germany. The FA-D1 cell lines were with biallelic mutations in the *BRCA2* gene (Table 1). All cell lines were cultured in RPMI 1640 medium (GIBCO BRL) with penicillin and streptomycin. 10% heat-inactivated fetal bovine serum (FBS, GIBCO BRL) was used for the *BRCA2 999del5* cell lines and the wild-type cell line whereas 10–20% non-heat inactivated FBS was used for the FA-D1 cell lines.

### 2.2. Chromosomal Analysis

Chromosomes were harvested after 3–4 h short-term culture with colcemid (0.016 μg/mL; KaryoMAX-colcemid, GIBCO, Patsley, UK). Telomere fluorescence in situ hybridization (FISH) was performed by ready-to-use Cy3-conjugated PNA pan-telomere probe (Telomere PNA FISH kit/Cy3, Cat. K5326, Dako, Glostrup, Denmark) in mixture with FITC-conjugated PNA pan-centromere probe (Dako) and counterstained with 4′,6-diamidino-2-phenylindole (DAPI) as previously described [9]. The microscope used for image capture was Leica DMRA2 connected to a Leica DC350F CCD camera and the Leica CW4000 image acquisition software. The image acquisition was performed through a ×100 oil objective by constant intensity and exposure times for different fluorophores. From each lymphoid cell line about 60 FISH labeled metaphases were analyzed. Events were counted on each metaphase and correction was made according to 46 chromosomes each metaphase if metaphases were aneuploidy. TL was detected either as a single telomere end (STE) on a single chromatid or as telomere free ends (TFE) on both sister chromatids. When results from STE and TFE analysis were combined the number of TFE was duplicated for both sister chromatids. Multitelomeric signals (MTS) were detected either as a single MTS (SMTS) on a sister chromatid or as double MTS (DMTS) on both sister chromatids. When results from SMTE and DMTS analysis were combined the number of DMTS was duplicated for both sister chromatids. ITS were detected as telomere FISH signals within a chromatid and ECTS as telomere FISH signals scattered around chromosomes without a centromere signal. The non-specific background noise of autofluorescence combined with red and green spots or cytoplasmic background were excluded and treated as unspecific background. Telomere FISH metaphase analysis was performed independently by four researchers in a blinded way for the genotypes of the EB lymphoid cell lines. Each *BRCA2*^+/-^ and *BRCA2*^-/-^ lymphoid cell line was analyzed by a single researcher. All researchers measured the same metaphase images of the EB0392 *BRCA2^+/+^* lymphoid cell line that was used as one of the controls and for correction of individual differences. The ECTS analysis was, however, performed by one researcher for all the lymphoid cell lines except one control line.

### 2.3. Statistical Analysis

Statistical analyses were performed on chromosomal abnormalities as per metaphase and results presented as an average mean with standard error of mean (SEM) bars. Results from each of the *BRCA2* genotypes were combined and performed as one genotype. A two-tailed Student´s *t*-test was used for statistical analysis and *p* < 0.05 was considered statistically significant. The R (CRAN) software was used for graphical representation.

## 3. Results

At least 60 metaphases were analyzed from each lymphoid cell line with one exception of the HSC62N cell line (Table 2). Most of the metaphases were diploid or near diploid with few exceptions of tetraploid metaphases or metaphases with incomplete chromosome number (Table 2, Supplementary Tables S1–S8).

In addition to analysis of TL, MTS, ITS and ECTS other chromosomal aberrations were measured including sister chromatid and end-to-end chromosome fusions, radial chromosomal configurations and chromosome fragments without telomere signals. These chromosomal aberrations were, however, not included in the results since average events per metaphase were below one which may affect the significance of the results.

### 3.1. Telomere Loss Was Found to Increase in a Stepwise Manner between the BRCA2 Genotypes

**Figure 1:**
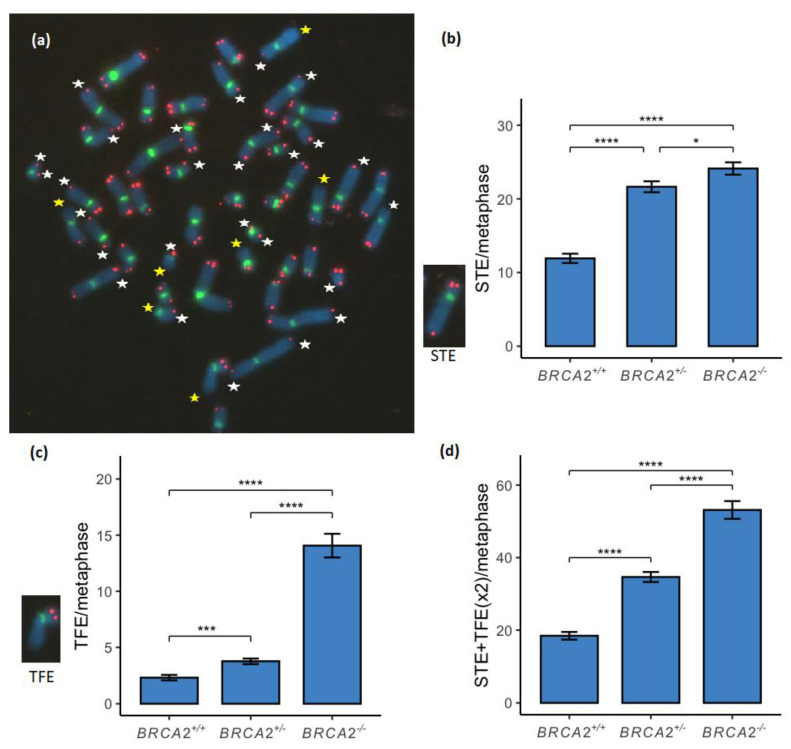
Telomere loss (TL) compared between the three *BRCA2* genotypes of lymphoid cell lines. (**a**) In each metaphase single telomere end (STE) was measured as a loss of a telomere signal (red) from a single sister chromatid (white stars) and telomere free ends (TFE) on chromosomes were measured when both telomere signals from a chromosome end were lost (yellow stars). (**b**) STE was significantly increased between the wild-type and the *BRCA2*^+/-^ genotypes and between the wild-type and the *BRCA2*^-/-^ genotypes (*p* < 0.0001), and significance was also found between the *BRCA2*^+/-^ and *BRCA2*^-/-^ genotypes (*p* < 0.05). (**c**) TFE was significantly increased between the three genotypes of *BRCA2*^+/+^, *BRCA2*^+/-^ and *BRCA2*^-/-^ in a stepwise manner (*p* < 0.001 and *p* < 0.0001, respectively). (**d**) TL was measured by a combination of numbers of STE and TFE duplicated. TL was significantly increased between the three genotypes of *BRCA2*^+/+^, *BRCA2*^+/-^ and *BRCA2*^-/-^ in a stepwise manner (*p* < 0.0001). * corresponds to *p* < 0.05, *** corresponds to *p* < 0.001 and **** corresponds to *p* < 0.0001. Metaphase chromosomes were analyzed with FISH by Cy3-conjugated telomere probe and FITC-conjugated centromere probe and counterstained with DAPI. The metaphase shown is from the EB1690 *BRCA2*^+/-^ lymphoid cell line.

TL was analyzed either as STE or TFE (Figure 1a). STE was found to be significantly increased in a stepwise manner between the *BRCA2^+/+^*, *BRCA2*^+/-^ and *BRCA2*^-/-^ lymphoid cell lines, and a significant difference was found between the *BRCA2*^+/-^ and *BRCA2*^-/-^ genotypes (Figure 1b; Supplementary Table S1). TFE was also found to be significantly increased in a stepwise manner between the *BRCA2* genotypes (Figure 1c; Supplementary Table S2). Note the strong difference found in TFE between the *BRCA2*^+/-^ and *BRCA2*^-/-^ genotypes, whereas a lower difference was found in STE between the two genotypes. Total TL was found to be significantly increased in a stepwise manner between the three *BRCA2* genotypes of lymphoid cell lines (Figure 1d; Supplementary Table S3).

### 3.2. Multitelomeric Signals Increase between BRCA2 Wild-Type and Mutated Genotypes

**Figure 2:**
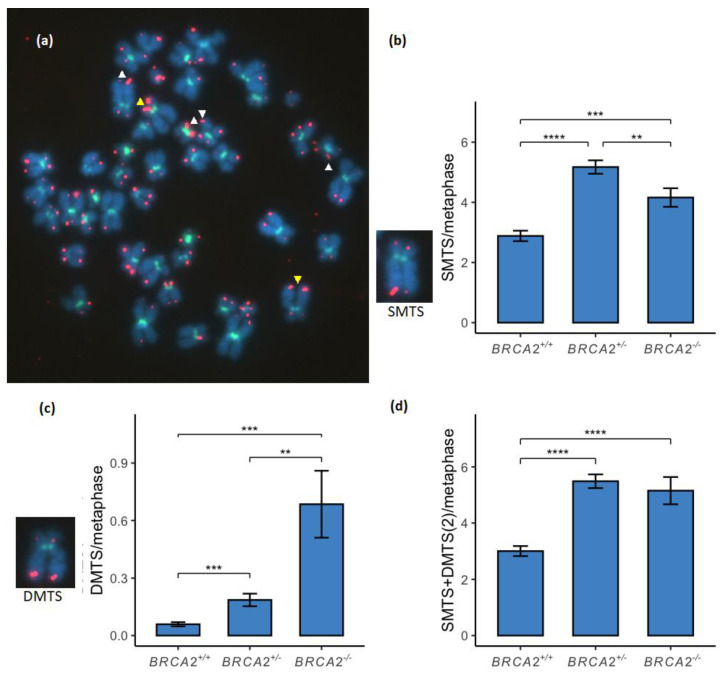
Multitelomeric signals (MTS) on chromosome ends compared between the three *BRCA2* genotypes of lymphoid cell lines. (**a**) Single-MTS (SMTS) on chromosome ends were measured as an MTS on a single sister chromatid (white triangles) and double-MTS (DMTS) on chromosome ends when both sister chromatids had MTS on a chromosome end (yellow triangles). (**b**) SMTS was significantly increased between the *BRCA2* wild-type and the *BRCA2*^+/-^ lymphoid cell lines (*p* < 0.0001) and the *BRCA2*^-/-^ lymphoid cell lines (*p* < 0.001). A significant difference was found between the *BRCA2*^+/-^ and *BRCA2*^-/-^ genotypes (*p* < 0.01). (**c**) DMTS were significantly increased between the three genotypes of *BRCA2*^+/+^, *BRCA2*^+/-^ and *BRCA2*^-/-^ in a stepwise manner (*p* < 0.001 and *p* < 0.01, respectively) and a significant difference was also found between the *BRCA2*^+/+^ and *BRCA2*^-/-^ genotypes (*p* < 0.001). (**d**) Total MTS were measured by a combination of numbers of SMTS and DMTS duplicated. MTS was significantly increased between the *BRCA2* wild-type, *BRCA2*^+/-^ and *BRCA2*^-/-^ genotypes (*p* < 0.0001). No difference was, however, found between the *BRCA2*^+/-^ and *BRCA2*^-/-^ genotypes in total MTS. ** corresponds to *p* < 0.01, *** corresponds to *p* < 0.001 and **** corresponds to *p* < 0.0001. Metaphase chromosomes were analyzed with FISH by Cy3-conjugated telomere probe and FITC-conjugated centromere probe and counterstained with DAPI. The metaphase shown is from the EB2302 *BRCA2*^+/-^ lymphoid cell line.

MTS were analyzed either as SMTS or DMTS (Figure 2a). SMTS was found to be significantly increased between the *BRCA2^+/+^* and the *BRCA2*^+/-^ lymphoid cell lines and between the *BRCA2^+/+^* and the *BRCA2*^-/-^ lymphoid cell lines (Figure 2b; Supplementary Table S4). SMTS was also found to be significantly higher among the *BRCA2*^+/-^ lymphoid cell lines compared to the *BRCA2*^-/-^ genotype. DMTS were, however, found to be significantly increased in a stepwise manner between the three *BRCA2* genotypes (Figure 2c; Supplementary Table S5). Total MTS was measured by a combination of SMTS and duplicated DMTS. Total MTS was found to be significantly increased between the *BRCA2* wild-type and the *BRCA2*^+/-^ lymphoid cell lines and between the *BRCA2* wild-type and the *BRCA2*^-/-^ lymphoid cell lines (Figure 2d; Supplementary Table S6). No difference of total MTS was, however, found between the two *BRCA2*^+/-^ and *BRCA2*^-/-^ genotypes.

### 3.3. Interstitial Telomere Sequences Increase in a Stepwise Manner between the BRCA2 Genotypes

**Figure 3:**
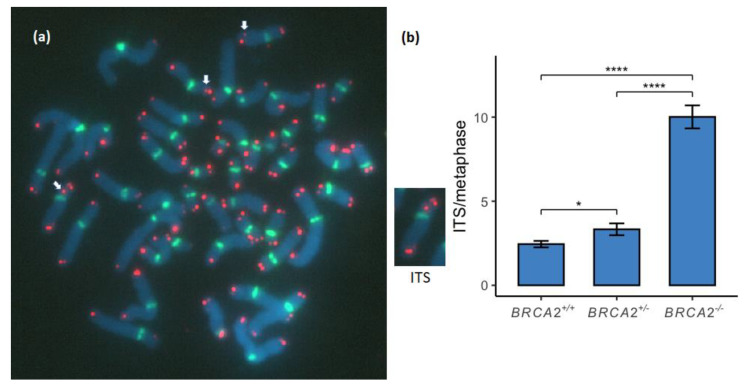
Interstitial telomere sequences (ITS) were compared between the *BRCA2* genotypes. (**a**) ITS were measured as signals within a chromosome arm (white arrows). (**b**) ITS were found to be significantly increased between the genotypes of *BRCA2*^+/+^, *BRCA2*^+/-^ and *BRCA2*^-/-^ in a stepwise manner (*p* < 0.05 and *p* < 0.0001, respectively). * corresponds to *p* < 0.05 and **** corresponds to *p* < 0.0001. Metaphase chromosomes were analyzed with FISH by Cy3-conjugated telomere probe and FITC-conjugated centromere probe and counterstained with DAPI. The metaphase shown is from the EB6085 *BRCA2*^+/-^ lymphoid cell line.

ITS were measured as telomere signals within chromosome arms (Figure 3a). ITS was found to be significantly increased between the *BRCA2* genotypes of the lymphoid cell lines in a stepwise manner (Figure 3b; Supplementary Table S7).

### 3.4. Extrachromosomal Telomere Sequences Increase in a Stepwise Manner between the BRCA2 Genotypes

**Figure 4:**
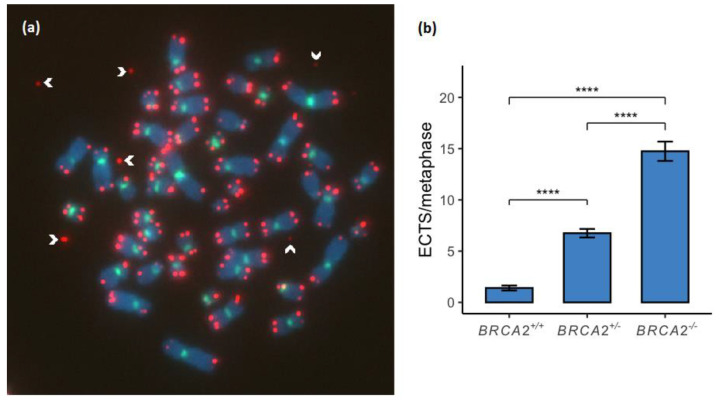
Extrachromosomal telomere sequences (ECTS) were compared between the *BRCA2* genotypes. (**a**) ECTS were measured as telomeric signals (red) scattered around the chromosomes (white arrowheads). (**b**) ECTS were found to be significantly increased between the genotypes of *BRCA2*^+/+^, *BRCA2*^+/-^ and *BRCA2*^-/-^ in a stepwise manner (*p* < 0.0001). **** corresponds to *p* < 0.0001. Metaphase chromosomes were analyzed with FISH by Cy3-conjugated telomere probe and FITC-conjugated centromere probe and counterstained with DAPI. The metaphase shown is from the EB2302 *BRCA2*^+/-^ lymphoid cell line.

ECTS were measured as DNA fragments with a telomere signal and without a centromere signal scattered around metaphases (Figure 4a). ECTS were found to be significantly increased between the *BRCA2* genotypes of the lymphoid cell lines in a stepwise manner (Figure 4b; Supplementary Table S8).

## 4. Discussion

TL is likely to result from RS that leads to terminal fork collapse, either at a single sister chromatid strand or at both sister chromatids on chromosome ends. TL was found to be increased among *BRCA2* heterozygous mammary epithelial cell lines in our previous study [9]. In the current study we found a significant stepwise increase in TL between the *BRCA2*^+/+^, *BRCA2*^+/-^ and *BRCA2*^-/-^ genotypes with a high difference between the *BRCA2*^+/+^ and *BRCA2*^+/-^ genotypes of the STE subgroup and between the *BRCA2*^+/-^ and *BRCA2*^-/-^ genotypes of the TFE subgroup (Figure 1). This indicates that events of TL caused by RS and collapsed terminal forks are BRCA2 haploinsufficiency dependent, showing an intermediate increase in TL with the STE subgroup and the most increase within the *BRCA2*^+/-^ genotype whereas the FA-D1 cell lines show most increase within the TFE subgroup. Terminal fork collapse can also appear as inter-chromatid discrepancies of telomere FISH signals [35]. Frequent telomere discrepancies were reported as unequal telomere signals on sister chromatids in our previous study on *BRCA2*^+/-^ mammary epithelial cell lines [9]. Telomere discrepancies were also frequently noted in the *BRCA2*^+/-^ and FA-D1 lymphoid cell lines in the present study although not reported. To further analyze telomere discrepancies in *BRCA2*^+/-^ cells and find out if they are consequences from terminal fork collapse or an ALT-related repair mechanism at the telomere termini or both, needs to be further analyzed by chromosome orientation FISH (CO-FISH) [36].

MTS is often referred to as fragile telomeres. They have been suggested to represent a recombination event among inter-/intra-chromatid telomeric sequences or ITS in the proximal regions of telomeres [37] or restarted collapsed terminal forks but their formation is still unknown. MTS has been shown to be suppressed by the key factors of HR repair, BRCA2 and RAD51 [18]. BRCA2 is required for telomere replication of the G-rich strand that has a high propensity to adopt G-quadruplex secondary structures leading to MTS formation in *BRCA2* deficient cells [38]. In the current study, we found a significant increase of MTS between the *BRCA2*^+/+^ and the *BRCA2*^+/-^ and *BRCA2*^-/-^ lymphoid cell lines, respectively (Figure 2). A significant increase of DMTS was also found between the *BRCA2*^+/-^ and *BRCA2*^-/-^ lymphoid cell lines but no difference was, however, found of the total MTS between the two genotypes. Lack of significance in MTS formation between the *BRCA2*^+/-^ and *BRCA2*^-/-^ genotypes may be explained by >50% higher TL of the *BRCA2*^-/-^ genotype (Figure 1c). The significant increase of DMTS found between the *BRCA2*^+/-^ and *BRCA2*^-/-^ genotypes is, however, a strong indication of BRCA2 deficiency related telomere RS due to G-quadruplex formation.

Most ITS probably result from the formation of DSB during RS that is mediated by targeted telomere insertions [35], although they have also been suggested to result from subsequent healing involving telomerase [39]. Some ITS may be the result of chromosome end-to-end fusions or chromatid fusions which can be detected as antiparallel orientated telomeres by CO-FISH. These ITS have been shown to be alternative TMEJ and include random nucleotides at the telomere junction [26]. Previously we reported significantly higher ITS frequency among *BRCA2*^+/-^ mammary epithelial cell lines compared to a commercial *BRCA2*^+/+^ mammary epithelial cell line and cell lines that rely on the classical ALT-positive mechanism for telomere maintenance [9]. In the present study we found a significant stepwise increase of ITS between the *BRCA2*^+/+^, *BRCA2*^+/-^ and *BRCA2*^-/-^ lymphoid cell lines (Figure 3) supporting our previous findings.

ECTS found scattered around chromosome spreads presumably result from terminal fork collapse caused by telomere RS. In this study we found a significant stepwise increase of ECTS between the *BRCA2*^+/+^, *BRCA2*^+/-^ and *BRCA2*^-/-^ lymphoid cell lines (Figure 4) probably directly correlated with increased TL between the three *BRCA2* genotypes. Sometimes these ECTS have strong fluorescence signals indicating amplification of telomeric c-circles that have been associated with cancer cells that rely on ALT rather than telomerase for telomere maintenance [40]. Recent studies have shown that abrogation of BRCA2 strongly increases c-circle amplification indicating the presence of ALT activity in BRCA2 deficient cells [30,41].

The results clearly show a gradual increase in telomere defects between the *BRCA2* genotypes. This was shown in lymphoid cells that are not known to be prone to *BRCA2* related cancer risk. It needs to be kept in mind, however, that apart from the *BRCA2* genotype the cell lines have generic individual genomic differences. For this same reason, the two *BRCA2*^+/+^ control cell lines used in this study may affect the interpretation. The most ideal setup would be to use a homogenous genetic background of a single cell line edited with the *CRISPR/Cas9* gene editing technique for insertion of the *BRCA2 9999del5* mutation. This has, however, not yet been successful due to the very low viability of cells with the homozygous mutation. This correlates with the fact that no homozygous individuals have been known to exist although expected under the Hardy–Weinberg equilibrium and allele frequency in the Icelandic population [5].

Our and other previous findings have shown unequal T-SCE in BRCA2 deficient cells [9,18,30]. Collapsed terminal forks can be recovered by T-SCE with two possible mechanisms, HR or BIR that is mediated by MiDAS [37]. HR dependent T-SCE is detected by DNA synthesis at both chromatid ends (semi-conservative DNA synthesis), whereas BIR dependent T-SCE is detected by DNA synthesis at single chromatid end (conservative DNA synthesis) by CO-FISH. Analysis by CO-FISH has shown that the ALT pathway in human cells can be a conservative DNA synthesis process potentially via the BIR pathway [42]. A more recent study showed that BRCA2 depletion reinforces RAD52 mediated BIR and engages with the ALT pathway by conservative telomeric DNA synthesis [30]. ALT has, however, been shown to be a bifurcated pathway involving both RAD52-dependent and RAD52-independent BIR. The RAD52-independent BIR pathway has been shown to be responsible for c-circle amplification which is reciprocally suppressed by RAD51 [43]. The HR-dependent semi-conservative DNA synthesis result as a response to Holliday junction resolution of the collapsed replication fork [37]. A recent study on the fruit fly showed that TMEJ compensates for the *BRCA2* dependent loss of HR on Holliday junction resolvases by using HR-intermediates that suppress mitotic crossing over and preserve the genomic stability [44]. BRCA2 deficiency has been shown to be synthetic lethal with disruption of either RAD52 or polymerase theta (POLQ) [26,27,45,46,47,48] or both [49]. Both RAD52 and POLQ are, therefore, important backup pathways for DSB repair and RS responses in BRCA2 deficient cells. RAD52 and POLQ are of high interest as therapeutic targets leading to synthetic lethal interaction of HR deficient tumors for future therapies especially due to acquired resistance of the currently used Poly (ADP-ribose) polymerase inhibitors [50]. However, the involvement of RAD52 and POLQ backup pathways in telomere maintenance of *BRCA2* deficient cells is not fully understood and needs to be further investigated.

Telomere length homeostasis in unaffected *BRCA2 999del5* mutation carriers does not differ from non-carriers in blood cells [19]. After a diagnosis of breast cancer, however, the measured relative telomere length in blood cells of *BRCA2 999del5* mutation carriers was found to be significantly shorter than among non-carriers. Telomere length was shown to be a modifier of breast cancer risk in *BRCA2 999del5* mutation carriers in the same study. Another aspect is that *BRCA2 999del5* mutation carriers with breast cancer have been associated with a significantly worse prognosis than non-carriers [51]. How defects in telomere length homeostasis of *BRCA2* mutation carriers might be related to consequences of possible telomere RS before and after breast cancer diagnosis needs to be answered in future studies. Such knowledge could have an impact on the prediction of early cancer development, and therefore, be used to advise the timing of preventive therapies, and targeted therapies of *BRCA2* related breast cancer in the future as discussed here above.

## 5. Conclusions

Taken together, we found a stepwise increase in telomere abnormalities between the *BRCA2*^+/+^, *BRCA2*^+/-^ and *BRCA2*^-/-^ genotypes showing a strong indication of BRCA2 haploinsufficiency in telomere maintenance. Our results from telomere FISH show a clear increase in TL, MTS, ITS and ECTS between the genotypes that are a sign of telomere RS in *BRCA2* deficient cells. More research is needed on the possible involvement of the RAD52 related BIR and the POLQ related TMEJ backup pathways in telomere maintenance when *BRCA2* is not fully expressed and the distinct roles of the two pathways on telomere maintenance and genomic stability.

## Figures and Tables

**Table 1:** List of lymphoid cell lines used in the study including information of the *BRCA2* genotype and mutation(s).

Lymphoid Cell Lines	*BRCA2* Genotype ^1^	*BRCA2* Mutation(s)	
EB0392	+/+	None	
EB6457	+/+	None	
EB1482	+/-	*999del5* ^2^	
EB1690	+/-	*999del5*	
EB1830	+/-	*999del5*	
EB2302	+/-	*999del5*	
EB6085	+/-	*999del5*	
HSC62N ^3^	-/-	IVS 19-1 G > A	IVS 19-1 G > A
NORD ^4^	-/-	886delGT	8447T > A
SPAN ^5^	-/-	15-16 exons del	1597del

^1^*BRCA2* genotypes: +/+ *BRCA2* wild-type, +/- *BRCA2* monoallelic mutation and -/- *BRCA2* biallelic mutations. ^2^ The official mutation name is rs80359671, NM_000059.3:c.767_771delCAAAT. ^3^ [13]. ^4^ Lymphoid cell line IFAR 772/1 from patient FA19 [33]. ^5^ Lymphoid cell line from patient FA62 [34].

**Table 2:** Metaphase number analyzed in each lymphoid cell line including information about mean chromosome number and the chromosome number range.

Lymphoid Cell Lines	*BRCA2* Genotype	Metaphases Analyzed	Mean Chromosome Number	Chromosome Number Range
EB0392	+/+	61	46.5	31–92
EB6457	+/+	65	45.4	31–83
EB1482	+/-	64	48.0	38–92
EB1690	+/-	60	47.6	41–92
EB1830	+/-	63	47.6	26–92
EB2302	+/-	62	46.6	45–92
EB6085	+/-	60	46.6	44–93
HSC62N	-/-	43	46.3	36–87
NORD	-/-	63	44.7	35–46
SPAN	-/-	66	45.0	39–47

## Data Availability

The data presented in this study are available in Supplementary Tables S1–S8.

## Supplementary Materials

The following supporting information can be downloaded at: https://www.mdpi.com/article/10.3390/genes13010083/s1, Table S1: Single telomere end (STE) analysis, Table S2: Analysis of telomere free ends (TFE), Table S3: Telomere loss (TL) as a combination of STE and TFE, Table S4: Analysis of multitelomere signals on single sister chromatids (SMTS), Table S5: Analysis of multitelomere signals on both (double) sister chromatids (DMTS), Table S6: Total multitelomere signals (MTS) by a combination of SMTS and DMTS, Table S7: Analysis of interstitial telomere sequences (ITS), Table S8: Analysis of extrachromosomal telomere sequences (ECTS).
